# Radial artery thrombus remodeling after transradial access: from fresh components to recanalized channels on OCT

**DOI:** 10.3389/fcvm.2026.1811724

**Published:** 2026-04-14

**Authors:** Haotian Wang, Jia Zhou, Saiying He, Hao Liu, Senhu Wang, Zixuan Li, Jincheng Guo

**Affiliations:** 1 Emergency Department, Beijing Luhe Hospital, Capital Medical University, Beijing, China; 2 Department of Cardiology, Beijing Luhe Hospital, Capital Medical University, Beijing, China

**Keywords:** optical coherence tomography (OCT), radial artery occlusion (RAO), radial artery thrombus (RAT), thrombus remodeling, transradial access (TRA)

## Abstract

**Background:**

Radial artery thrombus (RAT) is a key substrate of radial artery occlusion after transradial access (TRA), but *in vivo* phenotyping remains limited.

**Aims:**

This study aims to characterize RAT using optical coherence tomography (OCT), propose an OCT-based phenotyping scheme, and describe interval-stratified patterns after TRA.

**Methods:**

We retrospectively analyzed a prospectively maintained, single-center registry of consecutive radial artery OCT (RA-OCT) performed after OCT-guided coronary intervention (April 2019–September 2025). RAT was adjudicated by blinded readers using prespecified criteria: Type 1 (fresh), Type 2a/2b (non-recanalized without/with intrathrombus cavities), and Type 3 (recanalized with communicating channels). Phenotypes were compared across proximal, mid, and distal segments as well as across strata defined by time since the most recent ipsilateral TRA.

**Results:**

Eighteen patients underwent RA-OCT. The prevalence of Type 2b increased from proximal to distal segments (11.1% vs. 22.2% vs. 38.9%; *P* = 0.022), whereas Types 1 and 3 did not vary significantly across segments. Quantitative thrombus burden (thrombus length, minimal lumen area, and area stenosis) was similar across segments. With longer intervals since the most recent ipsilateral TRA, Type 1 decreased (*P* for trend=0.009) and Type 3 increased (*P* for trend = 0.014), with Type 3 predominating at ≥181 days.

**Conclusions:**

RA-OCT enables *in vivo* RAT phenotyping and supports a pragmatic OCT-based classification, demonstrating interval-dependent evolution toward a recanalized architecture after TRA.

## Introduction

Transradial access (TRA) has become the default approach for coronary angiography and percutaneous coronary intervention, but it is not without vascular sequelae. Among these, radial artery occlusion (RAO) remains one of the most common access-site complications (1). Accumulating clinical and imaging evidence indicates that radial artery thrombus (RAT) serves as the proximate pathophysiologic substrate for RAO (2), with the natural history of occlusion being dynamic: a substantial proportion of early occlusions recanalize spontaneously over time (3, 4). This pattern supports the concept that RAT undergoes time-dependent organization and remodeling, yet the *in vivo* morphologic correlates of this evolution remain incompletely characterized.

Ultrasound is the clinical standard for post-procedural assessment of radial artery patency because it evaluates vessel patency and flow, but it lacks the spatial resolution needed to characterize intraluminal thrombus microstructure (5–8). Consequently, mechanistic insight has relied largely on indirect inference from patency trajectories and on sparse, highly selected pathology (e.g., surgical specimens from severe hand ischemia or autopsy), which is unlikely to capture the full *in vivo* spectrum of thrombus phenotypes after TRA (7–10). To date, systematic *in vivo* OCT characterization of RAT has been scarce and largely confined to isolated reports describing features of thrombus organization (11–14).

Optical coherence tomography (OCT) provides near-histologic resolution and has transformed mechanistic phenotyping of coronary thrombosis, including the discrimination of fresh, organizing, and recanalized thrombus architectures (13–15). When applied to the radial artery, OCT offers a unique opportunity to directly visualize thrombus morphology and organization *in vivo*, moving beyond binary patency assessment toward phenotype-based characterization. Yet, a pragmatic and reproducible OCT-based phenotyping framework for RAT has not been established.

Accordingly, in this pilot study, we analyzed a consecutive series of radial artery OCT examinations and focused on the 18 patients with OCT-confirmed RAT and prior ipsilateral TRA to (1) delineate the *in vivo* OCT morphologic spectrum of RAT, (2) propose a pragmatic OCT-based phenotyping scheme, and (3) characterize interval-stratified phenotypes according to the time elapsed since the most recent ipsilateral TRA.

## Study design and population

We conducted a single-center retrospective analysis of a prospectively maintained radial artery OCT registry. At our center, radial artery OCT is routinely performed at the end of OCT-guided coronary intervention via TRA or distal TRA, including in patients undergoing repeat ipsilateral radial access after prior TRA. We retrospectively screened consecutive radial artery OCT examinations performed between April 2019 and September 2025. The final analytic cohort consisted of 18 patients with prior ipsilateral TRA in whom RAT was identified on OCT during repeat ipsilateral radial procedures, including both non-RAO repeat-access cases and RAO cases imaged after retrograde recanalization. Patients were excluded if radial artery OCT images were uninterpretable or of insufficient quality (e.g., poor blood clearance or severe artifacts) to permit adjudication. All participants provided written informed consent prior to cardiac catheterization. The study was approved by the institutional review board (IRB) of Beijing Luhe Hospital, Capital Medical University.

### RAO crossing and recanalization technique

In cases with angiographically confirmed RAO, retrograde recanalization was performed via distal radial access under fluoroscopic guidance using an operator-selected, stepwise wire strategy according to lesion resistance and crossing response. Microcatheter support, dottering, controlled knuckle wiring, and adjunctive lumen modification were used as needed to facilitate crossing or device advancement. Aspiration was not used. OCT was performed after completion of the coronary procedure. Detailed procedural characteristics of the four RAO cases are provided in Supplementary Table S1.

### Radial artery OCT acquisition

A 6F sheath (16 cm length; Terumo Co., Tokyo, Japan) was used in all patients. Distal TRA, when performed, was obtained at the anatomical snuffbox using the standard palpation-guided puncture technique at our center. Radial artery OCT was performed after completion of the coronary procedure according to our previously published protocol (16). The guiding catheter was removed while a coronary guidewire was maintained within the radial artery, and radial angiography was performed to localize the radial ostium. An external radiopaque ruler was positioned for length calibration. After intra-arterial administration of nitroglycerin (200 μg) and verapamil (2.5 mg), the sheath was retracted approximately 2 cm proximal to the puncture site, yielding an analyzable in-artery sheath length of approximately 14 cm. An external metallic marker (towel clamp) was placed at the skin entry site to identify the puncture location. The OCT catheter (C7XR or OPTIS; Abbott Vascular, Santa Clara, CA, USA) was advanced over the guidewire under fluoroscopic guidance to the radial ostium. To eliminate guidewire-related shadowing the guidewire was fully withdrawn and externalized (removed from the patient) after OCT catheter positioning and before image acquisition (Supplementary Figure S1). Supplementary Figure S1 provides a representative illustration of the rationale for guidewire withdrawal before image acquisition. Motorized pullbacks were performed at 20 mm/s during manual saline flush using pullback lengths of 54 mm (C7XR, Abbott Vascular) or 75 mm (OPTIS, Abbott Vascular). Based on the radiopaque ruler, adjacent pullbacks were aligned to provide an effective analyzed length of 50 mm for C7XR and 70 mm for OPTIS, corresponding to overlaps of approximately 4 and 5 mm, respectively.

### OCT-based definitions and quantitative measurements

Pullback start and analyzable segment length were determined from the fluoroscopic position of the OCT imaging lens relative to the radiopaque ruler and were confirmed by OCT longitudinal registration together with final radial artery angiography (Supplementary Figure S2), ensuring accurate segment matching and that overlapping regions were counted only once. OCT analyses were performed by two independent observers using an offline workstation (Abbott Vascular). Disagreements were resolved by consensus; when necessary, final adjudication was provided by an experienced OCT specialist. The overall OCT pullback length was divided into three segments based on sheath position after retraction: a proximal non-sheathed segment and two sheath-present segments (mid and distal, 7 cm each). The in-artery sheath length was approximately 14 cm. Thus, the proximal segment corresponded to the non-sheathed manipulation zone, whereas the distal segment additionally included the puncture/hemostasis region (Supplementary Figure S3).

### Thrombus definitions

**Figure 1:**
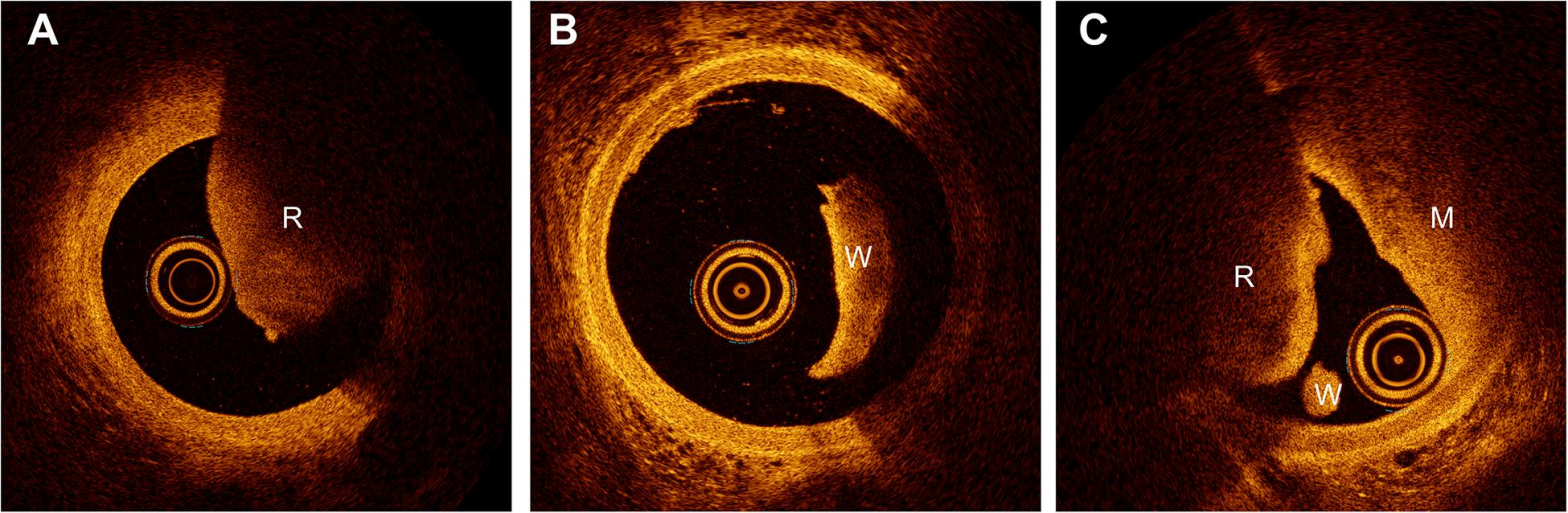
Fresh thrombus. **(A)** Red, white, and mixed thrombus within the lumen. **(B)** White thrombus observed in the lumen. **(C)** Red, white, and mixed thrombus. W, white; R, red; M, mixed.

**Figure 2:**
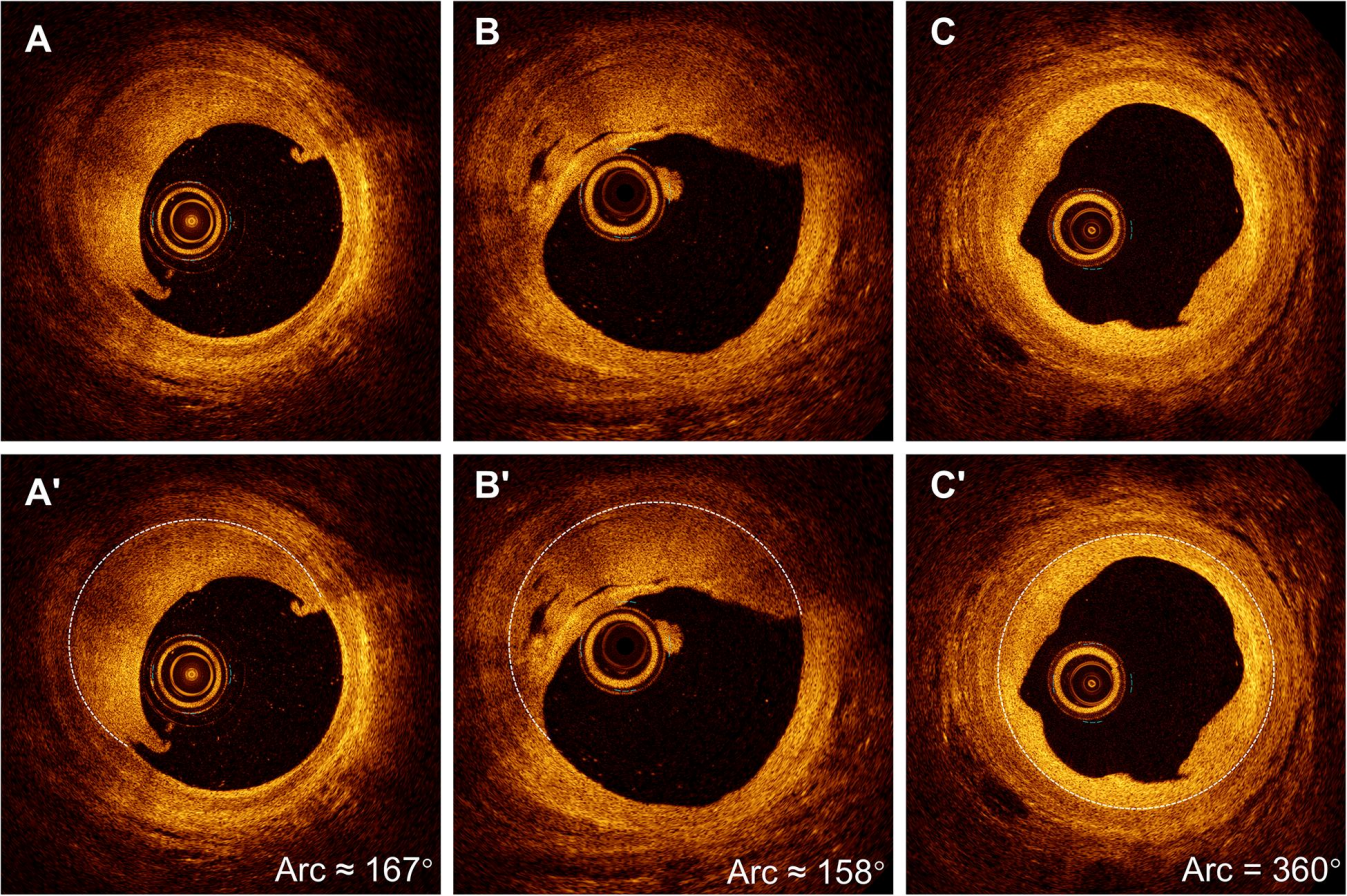
Non-recanalized thrombus without channels. **(A)** Layered thrombus without channels, with the arc shown in **(A′)**. **(B)** Thrombus with a cleft but without channels, with the corresponding arc shown in **(B′)**. **(C)** Irregular thrombus contour with homogeneous density, with the arc shown in **(C′)**.

**Figure 3:**
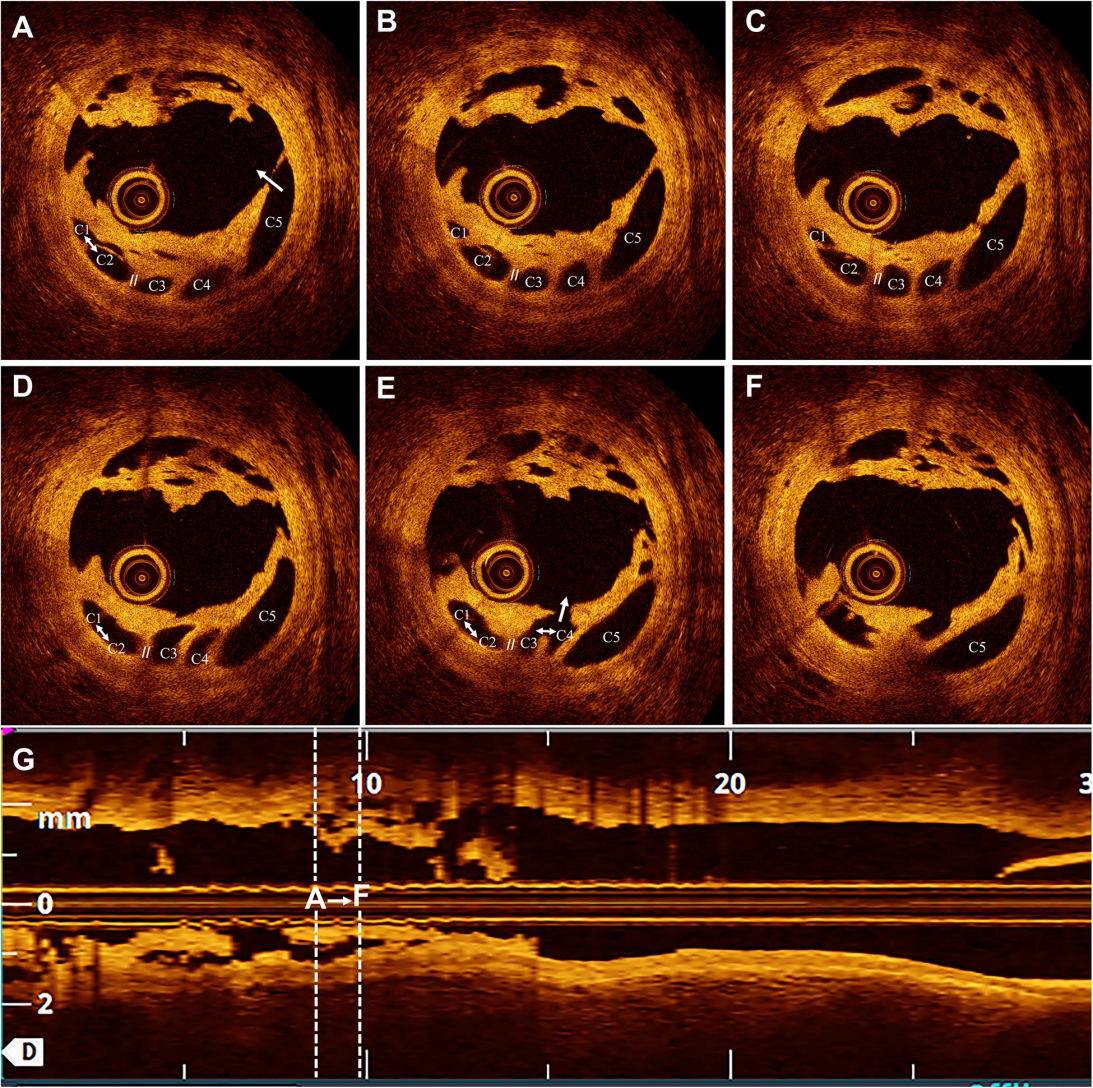
Non-recanalized thrombus with channels. Six consecutive frames showing thrombus with different channel configurations. **(A)** Channels C1–C5, with C1–C2 interconnected (↔); C5 communicates with the parent lumen (→). **(B,C)** Channels C1–C5 are non-interconnected (//). **(D)** Channels C1 and C2 are interconnected (↔). **(E)** Channels C1–C2 and C3–C4 are interconnected (↔) and communicate with the parent lumen (→). **(F)** Channel C5 is isolated and does not communicate with the parent lumen. **(G)** Longitudinal view corresponding to panels **(A–F)**.

**Figure 4:**
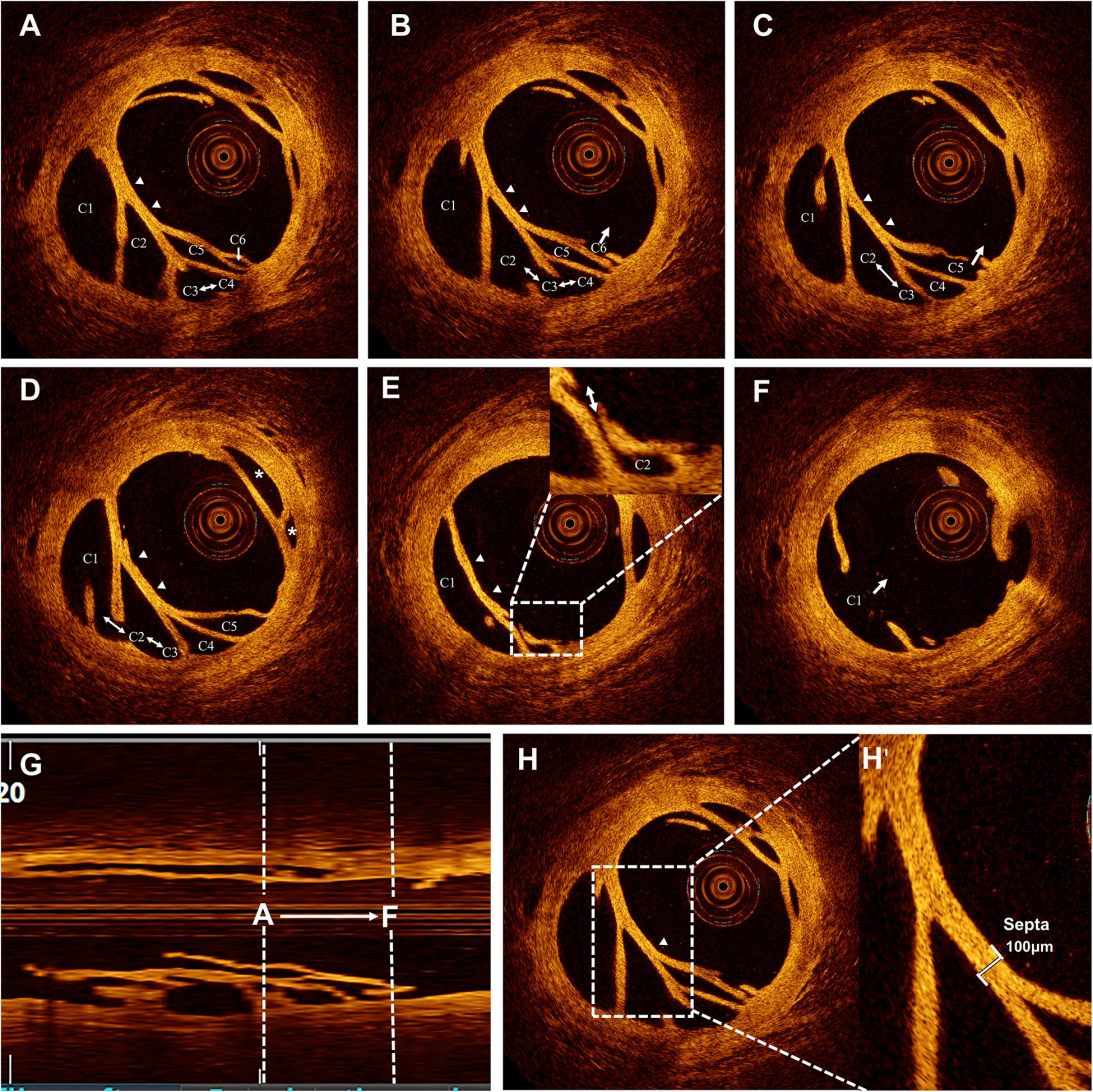
Recanalized thrombus with channels. Six channels (C1–C6) are shown with different configurations of interconnections and communication with the parent lumen. **(A)** Channels C3–C4 interconnected (↔). **(B)** Channels C2–C4 are interconnected (↔), with C6 communicating with the parent lumen (→). **(C)** Channels C2–C3 are interconnected (↔), with C5 communicating with the parent lumen (→). **(D)** Channels C1–C3 are interconnected (↔). **(E)** Channel C2 communicates with the parent lumen, magnified in the inset. **(F)** Channel C1 communicates with the parent lumen. **(G)** Longitudinal view corresponding to panels **(A–F)**. **(H)** Organized thrombus showing septa (arrowheads). **(H')** Magnified view of **H** showing thin septa (100 μm).

**Figure 5:**
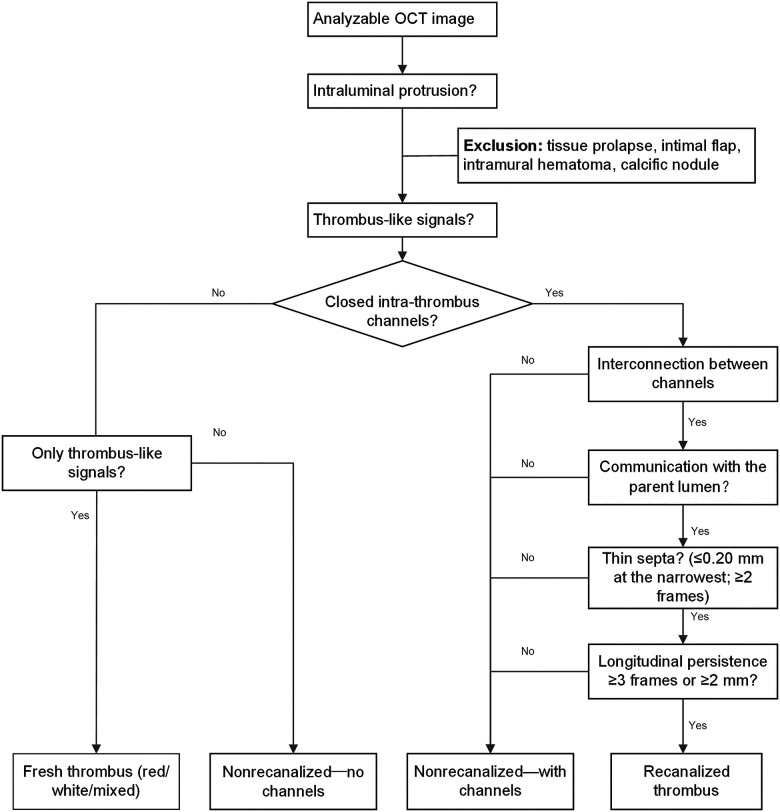
Thrombus classification flowchart. This flowchart classifies thrombus based on OCT characteristics, categorizing it as fresh, non-recanalized (with or without channels), or recanalized (defined by ≥3 interconnected channels, thin septa, and communication with the parent lumen).

Thrombus was classified on OCT as Type 1 (fresh), Type 2 (non-recanalized), or Type 3 (recanalized). Type 2 was further subtyped as Type 2a (without intrathrombus channels) and Type 2b (with intrathrombus channels that lack definite intercommunication and do not communicate with the parent lumen on serial frames; thus, they do not fulfill the criteria for Type 3). Representative examples are shown in Figures 1–4 and the Central Illustration. Type 1 was defined as an intraluminal thrombotic mass without enclosed intrathrombus cavities. In accordance with prior OCT consensus definitions, red thrombus was defined as a high-backscattering protrusion with signal-free shadowing, white thrombus was defined as a low-backscattering protrusion, and mixed thrombus was defined as coexisting red and white components within the same cross section (17–19). Type 3 (recanalized thrombus) was defined by longitudinally persistent, interconnected channels separated by thin septa, with definite interchannel communication and/or communication with the parent lumen, and was adjudicated only when these features were consistently observed on serial frames to minimize misclassification due to artifacts. Septal thickness for Type 3 was measured at the thinnest portion of the septum using a standardized approach: three measurements per frame were obtained and averaged, adapting established OCT methodology for fibrous cap thickness assessment (20–22). Figure 5 provides a schematic of the thrombus classification.

### Quantitative measurements

When multiple thrombus phenotypes coexisted within a given segment, segment-level quantitative summaries (“any thrombus”) were based on the phenotype with the greatest thrombus length. Thrombus length was measured using longitudinal reconstruction. Effective lumen area was defined differently by phenotype. For Type 1 and Type 2, effective lumen area reflected the true parent lumen and therefore excluded any enclosed intrathrombus cavities (Type 2b) that lacked definite communication with the parent lumen. For Type 3, each channel area was measured individually, and the effective lumen area was calculated as the sum of the areas of all communicating channels (i.e., channels with definite communication with the parent lumen). The minimum lumen area (MLA) was identified by segmental analysis at 1-mm intervals along the lesion. Percent area stenosis was calculated as (1 − MLA/reference lumen area) × 100%, where the reference lumen area was defined as the mean lumen area of the 5-mm proximal and 5-mm distal reference segments. Per cross-section channel metrics (Type 3) included (1) maximum channel count, (2) largest single-channel area, (3) minimum-to-maximum channel area ratio (restricted to cross sections with ≥2 channels), and (4) septa thickness.

### Study objectives

This study aimed to characterize the *in vivo* OCT-defined morphologic spectrum of RAT, with exploratory descriptive analyses according to the time since the most recent ipsilateral TRA and across radial artery segments.

### Statistical analysis

Continuous variables are presented as mean ± SD or median [interquartile range (IQR)], as appropriate, and categorical variables as *n*/*N* (%). Segment-level OCT metrics were analyzed as within-patient repeated measures across proximal, mid, and distal radial segments. For paired comparisons across segments, Cochran's *Q* test was used for binary variables and the Friedman test for continuous variables, using complete paired observations. For interval-stratified exploratory analyses according to the time since the most recent ipsilateral TRA (≤30, 31–180, 181–365, and >365 days), categorical variables were compared using trend-based methods and continuous variables using non-parametric tests, as appropriate. Comparisons of maximum channel count among patients with recanalized thrombus were performed only in strata with available observations. Given the sample size, all analyses were primarily descriptive and hypothesis-generating. All tests were two-sided, and *P* < 0.05 was considered nominally significant. *P*-values, including those in Supplementary Table S2, are exploratory and were not adjusted for multiplicity. Data were curated and tabulated using Microsoft Excel (Microsoft, Redmond, WA, USA.).

## Results

### Patient and procedural characteristics

Eighteen patients were included [age 61.8 ± 12.1 years; 14 men (77.8%)]. The median number of prior ipsilateral TRA procedures was 1.0 (IQR: 1.0–2.0), and the median interval from the most recent ipsilateral procedure to radial artery OCT was 182 days (IQR: 44–1,418). Distal TRA was used in 14 patients (77.8%). Angiographic RAO was present in four patients (22.2%); retrograde RAO recanalization was performed in these four cases, including three early after the most recent ipsilateral procedure [median 43 days (IQR: 39–52)] and one after a remote prior procedure (>4.5 years). Detailed procedural characteristics of these four RAO cases, including wire selection and crossing techniques, are provided in Supplementary Table S1. A typical case is shown in Supplementary Figure S4. Overall, six patients (33.3%) had ≥2 prior ipsilateral TRA procedures (Table 1).

**Table 1:** Baseline clinical and procedural characteristics.

Variable	Overall (*N* = 18)
Age (years)	61.8 ± 12.1
Male sex	14 (77.8)
Hypertension	13 (72.2)
Dyslipidemia	14 (77.8)
Diabetes mellitus	4 (22.2)
Current smoking	12 (66.7)
Prior PCI	8 (44.4)
Prior stroke	1 (5.6)
Peripheral artery disease	2 (11.1)
Chronic kidney disease	1 (5.6)
Carotid artery disease	4 (22.2)
Atrial fibrillation	1 (5.6)
Malignancy	1 (5.6)
Prior ipsilateral TRA, *n*	1.0 (1.0–1.8)
1 procedure	12 (66.7)
2 procedures	4 (22.2)
3 procedures	2 (11.1)
Days since the most recent ipsilateral TRA	182 (44–1,418)
Radial artery occlusion at enrollment	4 (22.2)
Distal radial access	14 (77.8)
Radial access	4 (22.2)
Index procedure: PCI	12 (66.7)
0.014-in. guidewire via radial artery	9 (50.0)
Diagnosis	
Unstable angina	13 (72.2)
STEMI	3 (16.7)
NSTEMI	2 (11.1)

PCI, percutaneous coronary intervention; PTCA, percutaneous transluminal coronary angioplasty; STEMI, ST-segment elevation myocardial infarction; NSTEMI, non-ST-segment elevation myocardial infarction; TRA, transradial access.

Values are mean ± SD, *n* (%), or median (interquartile range).

### Segmental OCT phenotypes and quantitative thrombus burden

Segment-level OCT findings are summarized in Table 2, Supplementary Table S3, and the Central Illustration. Reference vessel size did not differ significantly across segments (reference diameter *P* = 0.085; reference area *P* = 0.066). In contrast, phenotype prevalence varied by segment. Type 1 showed a distal enrichment trend [proximal 6/18 (33.3%), mid 8/18 (44.4%), distal 12/18 (66.7%); overall paired comparison *P* = 0.061], whereas Type 2b demonstrated distal enrichment [proximal 2/18 (11.1%), mid 4/18 (22.2%), distal 7/18 (38.9%); overall paired comparison *P* = 0.022]. Type 2a (*P* = 0.368) and Type 3 (*P* = 0.311) did not differ significantly across segments (Table 2, Supplementary Table S4).

**Table 2:** Segmental comparison of radial artery OCT findings.

Variable	Proximal	*n*	Mid	*n*	Distal	*n*	*P*-value
Reference vessel metrics							
Reference diameter (mm)	2.81 ± 0.44	18	2.66 ± 0.23	18	2.64 ± 0.29	18	0.085
Reference area (mm^2^)	6.36 ± 1.92	18	5.67 ± 0.88	18	5.54 ± 1.28	18	0.066
Phenotype prevalence, *n* (%)							
Type 1	6 (33.3)	18	8 (44.4)	18	12 (66.7)	18	0.061
Type 2a	2 (11.1)	18	2 (11.1)	18	3 (16.7)	18	0.368
Type 2b	2 (11.1)	18	4 (22.2)	18	7 (38.9)	18	0.022
Type 3	2 (11.1)	18	4 (22.2)	18	5 (27.8)	18	0.311
Any thrombus							
Thrombus length (mm)	36.0 (22.5–50.0)	7	35.6 (17.8–46.0)	11	21.6 (14.1–35.5)	16	0.819
Minimal lumen area (mm^2^)	2.85 (2.48–4.29)	7	3.65 (3.15–4.07)	11	4.06 (3.65–4.47)	16	0.607
Area stenosis (%)	54.34 (42.96–60.41)	7	31.30 (25.48–40.19)	11	22.55 (15.62–44.73)	16	0.115

OCT, optical coherence tomography.

Values are mean ± SD, median (interquartile range), or *n* (%). *P*-values compare proximal, mid, and distal segments using paired analyses where applicable.

**Central Illustration:**
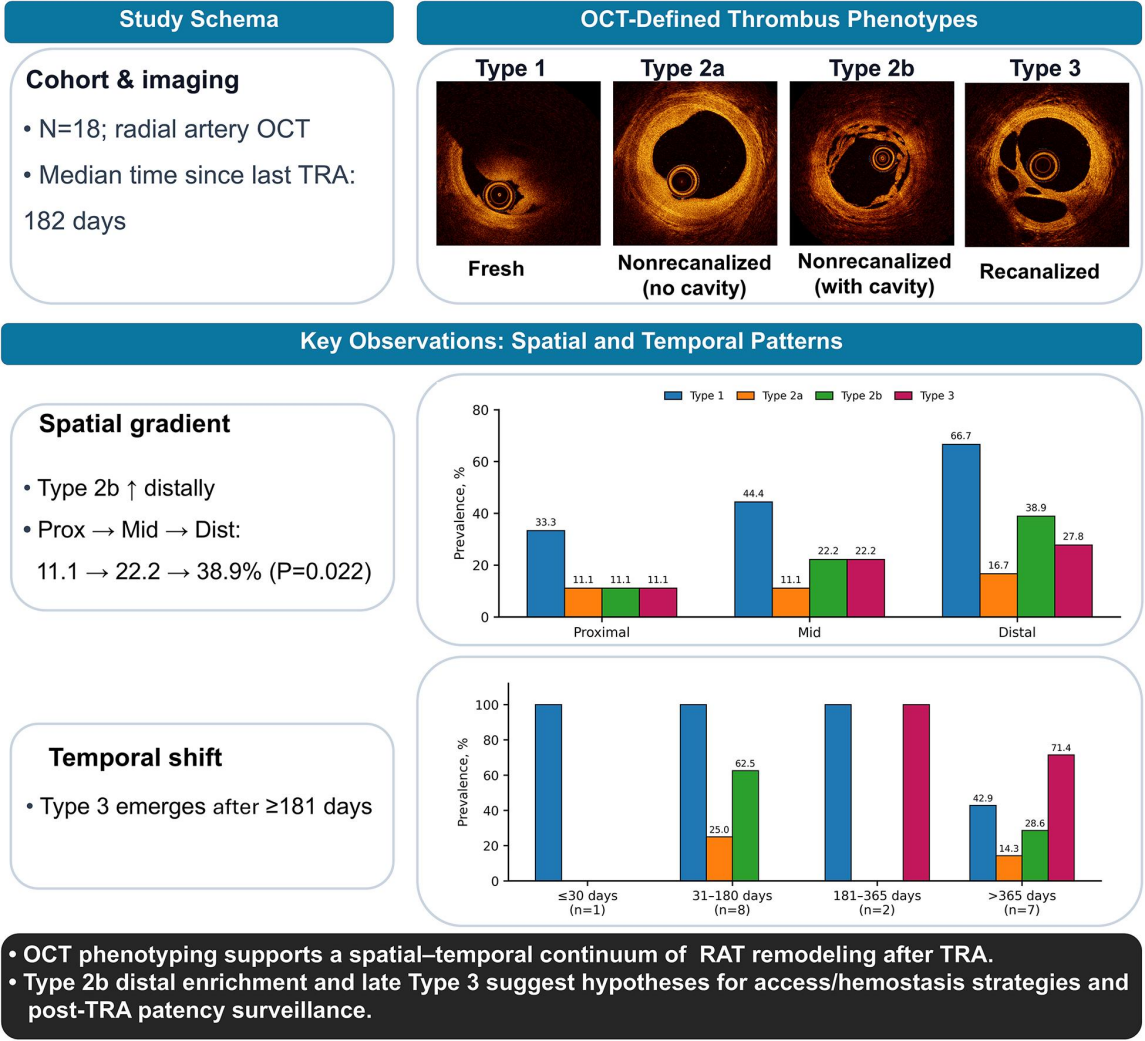
Spatial–temporal remodeling of radial artery thrombus after TRA. In 18 patients undergoing radial artery OCT (median 182 days since the most recent ipsilateral transradial access), thrombus phenotypes were classified as Type 1 (fresh), Type 2a (non-recanalized without cavity), Type 2b (non-recanalized with cavity), and Type 3 (recanalized). Type 2b showed distal enrichment (proximal 11.1% → mid 22.2% → distal 38.9%; *P* = 0.022). Interval stratification suggested later-stage remodeling, with Type 3 observed at ≥181 days. OCT, optical coherence tomography; RA, radial artery; TRA, transradial access.

### Interval-stratified phenotypes

Phenotype prevalence varied with time since the most recent ipsilateral TRA (Table 3, Supplementary Table S2). Type 1 decreased across increasing time-interval strata (*P* for trend = 0.011), whereas Type 3 was not observed within 180 days and emerged thereafter (*P* for trend = 0.004). In exploratory analyses, total thrombus length (summed across segments and phenotypes) differed across time strata (*P* = 0.053) (Supplementary Table S2).

**Table 3:** Interval-stratified phenotypes by time since the most recent TRA.

Variable	Overall (*N* = 18)	≤30 days (*n* = 1)	31–180 days (*n* = 8)	181–365 days (*n* = 2)	>365 days (*n* = 7)
Days since the most recent TRA (days)	182 (44–1,418)	2 (2–2)	44 (38–48)	266 (235–298)	1,786 (1,270–3,308)
Any thrombus (any phenotype)	18 (100.0)	1 (100.0)	8 (100.0)	2 (100.0)	7 (100.0)
Thrombus phenotype prevalence, *n* (%)					
Type 1	14 (77.8)	1 (100.0)	8 (100.0)	2 (100.0)	3 (42.9)
Type 2a	3 (16.7)	0 (0.0)	2 (25.0)	0 (0.0)	1 (14.3)
Type 2b	7 (38.9)	0 (0.0)	5 (62.5)	0 (0.0)	2 (28.6)
Type 3	6 (33.3)	0 (0.0)	0 (0.0)	2 (100.0)	4 (57.1)
Type 3 characteristics					
Maximum number of channels	5.0 (4.5–8.0)	—	—	5.5 (4.2–6.8)	5.0 (5.0–8.0)
Total thrombus length (mm)	48.3 (29.4–105.0)	29.0 (29.0–29.0)	117.4 (69.5–191.4)	48.5 (42.5–54.5)	18.8 (13.6–48.3)
RAO at enrollment, *n* (%)	4 (22.2)	0 (0.0)	3 (37.5)	0 (0.0)	1 (14.3)

TRA, transradial access; RAO, radial artery occlusion.

Values are median (interquartile range) or *n* (%). Interval categories are defined by the number of days elapsed since the most recent ipsilateral TRA. Channel-related metrics are reported only for patients with recanalized (Type 3) thrombus.

## Discussion

Two major findings emerged. First, radial artery OCT enabled *in vivo* phenotyping of RAT and supported a pragmatic OCT-based classification (Types 1, 2a/2b, and 3). Second, phenotype distribution was interval-dependent after the most recent ipsilateral TRA, with Type 3 predominantly observed at later intervals (approximately beyond 6 months), consistent with progressive thrombus remodeling.

### Methods to identify RAT

RAT can be evaluated by angiography, duplex ultrasound, histopathology, and OCT; however, accurate *in vivo* identification and staging remain challenging. Angiographic findings (e.g., haziness or filling defects) are non-specific (23, 24). Duplex ultrasound confirms patency and assesses flow limitation but has limited resolution for small mural thrombi and microstructural features such as microchannels and thin septa (25, 26). Histopathology is the reference standard for thrombus composition and maturity but is rarely available and non-repeatable (8, 27). In contrast, OCT provides near-histologic *in vivo* visualization of organized/recanalized architecture—channels, septa, and intercommunications—supporting phenotype-based characterization (19, 28).

### OCT-based phenotypes of RAT

Prior intracoronary OCT studies have delineated a thrombus spectrum from fresh thrombus to layered/healing phenotypes and ultimately to organized thrombus with recanalization channels communicating with the parent lumen (29–32). In contrast, RAT has often been assessed immediately after PCI, where thrombi are predominantly fresh (12), whereas later organized or recanalized appearances have been reported only in isolated case reports without a standardized framework (33, 34). Accordingly, classifications spanning early through recanalized stages remain insufficiently established for RAT.

Using systematic radial OCT in 18 patients imaged across heterogeneous time intervals, we developed a pragmatic OCT-based classification (Types 1–3, with prespecified subtypes) to standardize major morphologic phenotypes and enable interval-stratified analyses. This framework is not intended to “date” thrombus precisely, particularly in advanced organization where collagen densification may reduce tissue contrast (30). Rather, it provides a common language to facilitate cross-study comparisons and hypothesis generation.

Prior pathology studies support a time-dependent thrombus continuum, with early lytic changes over days progressing to organization after approximately 1 week (35, 36), followed by neovascularization and recanalization with multiple endothelialized channels evolving over weeks to months (19). Contemporary OCT descriptions of honeycomb-/lotus root-like patterns are consistent with recanalized, organized thrombus (37). In our cohort, Type 1 was enriched early (approximately within 2 months), whereas Type 3 appeared only at later intervals (approximately from 6 months onward) and presented as a pure honeycomb-/lotus root-like pattern without concomitant Type 1/2 features. Type 2a/2b predominated early and frequently coexisted within the same patient, underscoring substantial morphologic heterogeneity during thrombus remodeling.

### Spatial distribution and segmental phenotypes of thrombus

Our segment-level analysis suggests that thrombus phenotype is not spatially uniform along the radial artery. Despite comparable reference vessel caliber across segments, Type 2b was significantly more prevalent distally, whereas Type 2a and Type 3 did not differ by segment. This phenotype–location discordance (despite similar caliber) supports a role for local mechanical and hemodynamic determinants rather than anatomy alone, particularly within sheath-instrumented segments where OCT has demonstrated frequent intimal injury after transradial instrumentation (13). The distal segment is additionally exposed to puncture-site compression and low-flow/stasis during hemostasis, and the RAO prevention literature on patent hemostasis supports a flow-mediated contribution to thrombotic remodeling (38–40).

Among cases with complete three-segment paired data, thrombus burden metrics (length, MLA, and area stenosis) were similar, whereas Type 3 channel architecture was consistent with canonical OCT descriptions, underscoring the limited specificity of angiographic “hazy/fuzzy” appearances. However, segment-level comparisons of recanalization descriptors remained underpowered due to limited complete pairing and small sample size (19, 23).

### Integrated spatial–temporal framework

Integrating segment-level and interval-stratified analyses, thrombus phenotype varied by time and location: Type 3 was observed only at later intervals, whereas Type 2b showed distal predominance despite similar vessel size and thrombus burden across segments. Collectively, these findings support a hypothesis-generating spatial–temporal remodeling continuum, in which early non-recanalized phenotypes may transition through Type 2b as an intermediate state and, in a subset of cases, progress to Type 3 over time.

### Clinical implications for radial preservation and RAO recanalization

In patients with prior ipsilateral TRA, resistance to guidewire advancement after successful radial/distal radial puncture should prompt consideration of access-artery thrombosis or RAO, in addition to spasm or anatomic impediments (41). Forceful manipulation should be avoided. The mechanism should be clarified (e.g., with radial angiography), and any crossing—if pursued—should follow a morphology-guided wire strategy under fluoroscopic guidance. In our RAO cases, this commonly required polymer-jacketed wires, controlled knuckle wiring, and adjunctive lumen modification. Accordingly, wire selection should be guided by lesion morphology and crossing resistance, whereas more organized or complete occlusions may require escalation to hydrophilic or polymer-jacketed wires and drilling or controlled knuckle techniques (42).

Retrograde RAO recanalization (often via distal TRA) has been reported to achieve high procedural success across series and registries (42–45). In our cohort, post-recanalization OCT most often demonstrated non-recanalized morphologies (predominantly Type 2a/2b, occasionally with residual Type 1), with mature Type 3 observed infrequently—particularly given that three of four cases were imaged relatively early after the most recent ipsilateral TRA (39–52 days). These observations suggest that, in some lesions, thrombus organization may not yet be fully mature at the time of reopening. This may be one possible explanation for the feasibility of guidewire crossing in some RAO lesions, but it should be considered hypothesis-generating within the scope of the present imaging study. Because this registry included both routine repeat-access cases and RAO cases imaged after deliberate recanalization, these findings should not be interpreted as defining the natural history of spontaneous recanalization over time.

### Limitations

Several limitations warrant consideration. First, this was a small, single-center retrospective study, limiting statistical power, generalizability, and causal inference. Second, segment-level comparisons required complete proximal–mid–distal data and therefore resulted in variable effective sample sizes across analyses; recanalization microstructural descriptors were consequently reported descriptively due to limited observations. Third, OCT phenotyping lacked histopathologic validation and should be interpreted as a morphologic framework rather than a precise temporal “dating” system. Fourth, because radial OCT was performed after completion of the coronary procedure, sheath or catheter manipulation and, in some cases, deliberate RAO crossing or recanalization may have modified thrombus microstructure and influenced OCT-based phenotyping, including the discrimination between Type 2b cavities and mature Type 3 recanalization. Finally, the interval was indexed to the most recent ipsilateral TRA, which may not capture the cumulative effects of earlier access events in patients with multiple prior procedures. Accordingly, these findings are hypothesis-generating and warrant confirmation in larger prospective cohorts with standardized segment acquisition, longitudinal follow-up, and—where feasible—pathologic correlation and clinical outcome linkage.

## Conclusions

RAT phenotype is spatially heterogeneous along the artery. A distal predominance of Type 2b morphology was observed despite similar vessel size and thrombus burden across segments, suggesting segment-specific differences in thrombus organization. Phenotypes also shifted over time, with recanalized patterns becoming predominant beyond approximately 6 months after the most recent ipsilateral TRA. Collectively, these findings highlight the dynamic and heterogeneous nature of RAT remodeling after TRA.

## Data Availability

The original contributions presented in the study are included in the article/Supplementary Material; further inquiries can be directed to the corresponding author.

## References

[1] Sandoval Y Bell MR Gulati R. Transradial artery access complications. Circ: Cardiovasc Interv. (2019) 12(11):e007386. 10.1161/CIRCINTERVENTIONS.119.007386 31672030

[2] Didagelos M Afendoulis D Pagiantza A Moysidis D Papazoglou A Kakderis C Treatment of radial artery occlusion after transradial coronary catheterization: a review of the literature and proposed treatment algorithm. Hellenic J Cardiol. (2025) 84:81–95. 10.1016/j.hjc.2025.01.008 39909225

[3] Sinha SK Jha MJ Mishra V Thakur R Goel A Kumar A Radial artery occlusion—incidence, predictors and long-term outcome after transradial catheterization: clinico-Doppler ultrasound-based study (Rail-Trac Study). Acta Cardiol. (2017) 72(3):318–27. 10.1080/00015385.2017.1305158 28636520

[4] Garg N Madan BK Khanna R Sinha A Kapoor A Tewari S Incidence and predictors of radial artery occlusion after transradial coronary angioplasty: Doppler-guided follow-up study. J Invasive Cardiol. (2015) 27(2):106–12. 25661763

[5] Avdikos G Karatasakis A Tsoumeleas A Lazaris E Ziakas A Koutouzis M. Radial artery occlusion after transradial coronary catheterization. Cardiovasc Diagn Ther. (2017) 7(3):305–16. 10.21037/cdt.2017.03.14 28567356 PMC5440258

[6] Tsigkas G Papanikolaou A Apostolos A Kramvis A Timpilis F Latta A Preventing and managing radial artery occlusion following transradial procedures: strategies and considerations. J Cardiovasc Dev Dis. (2023) 10(7):283. 10.3390/jcdd10070283 37504539 PMC10380353

[7] Cederholm I Sørensen J Carlsson C. Thrombosis following percutaneous radial artery cannulation. Acta Anaesthesiol Scand. (1986) 30(3):227–30. 10.1111/j.1399-6576.1986.tb02402.x 3739580

[8] Chitte SA Veltri K Thoma A. Ischemia of the hand secondary to radial artery thrombosis: a report of three cases. Can J Plast Surg. (2003) 11(3):145–8. 10.1177/229255030301100308 24115858 PMC3792752

[9] Bengezi OA Dalcin A Al-Thani H Bain JR. Unusual complication of radial artery cannulation. Can J Plast Surg. (2003) 11(4):213–5. 10.1177/229255030301100409 24009442 PMC3760753

[10] Garg K Howell BW Saltzberg SS Berland TL Mussa FF Maldonado TS Open surgical management of complications from indwelling radial artery catheters. J Vasc Surg. (2013) 58(5):1325–30. 10.1016/j.jvs.2013.05.011 23810262

[11] Liu ZJ Niu D Li ZX Guo JC. Radial artery thrombosis in optical coherence tomography guided transradial coronary angiography and percutaneous coronary intervention in acute coronary syndrome patients and its risk factors analysis. Zhonghua Xin Xue Guan Bing Za Zhi. (2021) 49(1):37–42. 10.3760/cma.j.cn112148-20200312-00196 33429484

[12] Liu Z Wang G Niu D Wu Y Li Z Zhang L Bivalirudin vs. heparin on radial artery thrombosis during transradial coronary intervention: an optical coherence tomography study. J Interv Cardiol. (2020) 2020:1. 10.1155/2020/7905021 PMC7533783 33071677

[13] Yonetsu T Kakuta T Lee T Takayama K Kakita K Iwamoto T Assessment of acute injuries and chronic intimal thickening of the radial artery after transradial coronary intervention by optical coherence tomography. Eur Heart J. (2010) 31(13):1608–15. 10.1093/eurheartj/ehq102 20413398

[14] Novakova T Kanovsky J Miklik R Bocek O Poloczek M Jerabek P Short sheath benefit in radial artery injury after PCI—optical coherence tomography serial study. Biomed Pap. (2016) 160(3):393–8. 10.5507/bp.2016.035 27641357

[15] Eriksen E Herstad J Pertiwi KR Tuseth V Nordrehaug JE Bleie O Thrombus characteristics evaluated by acute optical coherence tomography in ST elevation myocardial infarction. PLoS One. (2022) 17(4):e0266634. 10.1371/journal.pone.0266634 35404941 PMC9000063

[16] Wang Y Yan R Li Z Liu Z Wang Y Song J Mapping the distribution of radial artery atherosclerosis by optical coherence tomography. BMC Med Imag. (2025) 25(1):47. 10.1186/s12880-025-01583-7 PMC11827234 39948453

[17] Yonetsu T Jang IK. Cardiac optical coherence tomography: history, current status, and perspective. JACC: Asia. (2024) 4(2):89–107. 10.1016/j.jacasi.2023.10.001 38371282 PMC10866736

[18] Toutouzas K Karanasos A Stathogiannis K Synetos A Tsiamis E Papadopoulos D A honeycomb-like structure in the left anterior descending coronary artery: demonstration of recanalized thrombus by optical coherence tomography. JACC Cardiovasc Interv. (2012) 5(6):688–9. 10.1016/j.jcin.2012.01.024 22721666

[19] Kang SJ Nakano M Virmani R Song HG Ahn JM Kim WJ Oct findings in patients with recanalization of organized thrombi in coronary arteries. JACC: Cardiovasc Imaging. (2012) 5(7):725–32. 10.1016/j.jcmg.2012.03.012 22789941

[20] Fujii K Kubo T Otake H Nakazawa G Sonoda S Hibi K Expert consensus statement for quantitative measurement and morphologic assessment of optical coherence tomography: update 2025. Cardiovasc Interv Ther. (2025) 40(2):226–33. 10.1007/s12928-024-01080-8 39873844 PMC11910418

[21] Tearney GJ Regar E Akasaka T Adriaenssens T Barlis P Bezerra HG Consensus standards for acquisition, measurement, and reporting of intravascular optical coherence tomography studies: a report from the international working group for intravascular optical coherence tomography standardization and validation. J Am Coll Cardiol. (2012) 59(12):1058–72. 10.1016/j.jacc.2011.09.079 22421299

[22] Araki M Park SJ Dauerman HL Uemura S Kim JS Di Mario C Optical coherence tomography in coronary atherosclerosis assessment and intervention. Nat Rev Cardiol. (2022) 19(10):684–703. 10.1038/s41569-022-00687-9 35449407 PMC9982688

[23] Souteyrand G Valladier M Amabile N Derimay F Harbaoui B Leddet P Diagnosis and management of spontaneously recanalized coronary thrombus guided by optical coherence tomography—lessons from the French “lotus root” registry. Circ J. (2018) 82(3):783–90. 10.1253/circj.CJ-17-0810 29199266

[24] Xu T Shrestha R Pan T Huang X Xu H Zhang JJ Anatomical features and clinical outcome of a honeycomb-like structure in the coronary artery: reports from 16 consecutive patients. Coron Artery Dis. (2020) 31(3):222–9. 10.1097/MCA.0000000000000822 31658133

[25] Koy Ay E Naldemir IF Ozde C Akture G Aytekin S Kayapinar O Radial artery thrombosis and associated risk factors in patients undergoing radial coronary angiography. Heart Int. (2024) 18(2):37–43. 10.17925/HI.2024.18.2.1 39885935 PMC11781368

[26] Maadani M Ardestani SS Rafiee F Rezaei-Kalantari K Rabiee P Kia YM Rivaroxaban versus enoxaparin in patients with radial artery occlusion after transradial coronary catheterization: a pilot randomization trial. Vasc Specialist Int. (2025) 41:2. 10.5758/vsi.240103 39994502 PMC11850656

[27] Bulguroglu S Calapkulu Y Koc U Erdogan M Golbasi Z. C-reactive protein to albumin ratio and radial artery thrombosis post transradial angiography. Biomark Med. (2024) 18(9):471–78. 10.1080/17520363.2024.2345578 PMC11285348 39007835

[28] Gupta A Rao KR Reddy SS Kashyap JR Kadiyala V Kaur J Optical coherence tomography characterization of spontaneous recanalized coronary thrombus—single center experience. J Cardiovasc Thorac Res. (2022) 14(4):220–7. 10.34172/jcvtr.2022.30504 36699554 PMC9871162

[29] Kume T Akasaka T Kawamoto T Ogasawara Y Watanabe N Toyota E Assessment of coronary arterial thrombus by optical coherence tomography. Am J Cardiol. (2006) 97(12):1713–7. 10.1016/j.amjcard.2006.01.031 16765119

[30] Shimokado A Matsuo Y Kubo T Nishiguchi T Taruya A Teraguchi I *In vivo* optical coherence tomography imaging and histopathology of healed coronary plaques. Atherosclerosis. (2018) 275:35–42. 10.1016/j.atherosclerosis.2018.05.025 29859471

[31] Araki M Yonetsu T Russo M Kurihara O Kim HO Shinohara H Predictors for layered coronary plaques: an optical coherence tomography study. J Thromb Thrombolysis. (2020) 50(4):886–94. 10.1007/s11239-020-02116-5 32306291

[32] Kato M Dote K Sasaki S. Recanalized image of thrombotic occlusion with coronary plaque rupture: a lotus root-like appearance by optical coherence tomography. Can J Cardiol. (2011) 27(6):871e1–2. 10.1016/j.cjca.2011.08.125 22014625

[33] Kim S Lee SY Kim Y Lee DI Lee JH Bae JW Optical coherent tomographic (OCT) finding of radial arterial recanalization. Korean Circ J. (2020) 50(11):1045–7. 10.4070/kcj.2020.0110 32725994 PMC7596215

[34] Yoshioka G Sonoda S Nagao M Node K. Lotus root-like appearance at the radial artery in a Buerger-like disease patient. JACC: Cardiovasc Interv. (2023) 16(17):2183–4. 10.1016/j.jcin.2023.06.036 37565969

[35] Kramer MC Rittersma SZ de Winter RJ Ladich ER Fowler DR Liang YH Relationship of thrombus healing to underlying plaque morphology in sudden coronary death. J Am Coll Cardiol. (2010) 55(2):122–32. 10.1016/j.jacc.2009.09.007 19818571

[36] Alkarithi G Duval C Shi Y Macrae FL Ariëns RAS. Thrombus structural composition in cardiovascular disease. Arterioscler Thromb Vasc Biol. (2021) 41(9):2370–83. 10.1161/ATVBAHA.120.315754 34261330 PMC8384252

[37] Quevedo F Farjat J Bertrand O Poulin A Dery JP Garcia-Labbe D Honeycomb or lotus root-like intracoronary pattern: insights from optical coherence tomography of a recanalized thrombus. Catheter Cardiovasc Interv. (2025) 106(4):2511–18. 10.1002/ccd.70089 40798907 PMC12502023

[38] Bernat I Aminian A Pancholy S Mamas M Gaudino M Nolan J Best practices for the prevention of radial artery occlusion after transradial diagnostic angiography and intervention: an international consensus paper. JACC: Cardiovasc Interv. (2019) 12(22):2235–46. 10.1016/j.jcin.2019.07.043 31753298

[39] Kyriakopoulos V Xanthopoulos A Papamichalis M Skoularigkis S Tzavara C Papadakis E Patent hemostasis of radial artery: comparison of two methods. World J Cardiol. (2021) 13(10):574–84. 10.4330/wjc.v13.i10.574 34754402 PMC8554357

[40] Eid-Lidt G Reyes-Carrera J Farjat-Pasos JI Saenz AL Bravo CA Rangel SN Prevention of radial artery occlusion of 3 hemostatic methods in transradial intervention for coronary angiography. JACC: Cardiovasc Interv. (2022) 15(10):1022–9. 10.1016/j.jcin.2022.03.011 35589232

[41] Bland AC Meere W Mikhail P Chuah E Redwood E Ferreira D Enhancing guidewire efficacy for transradial access: the EAGER randomized controlled trial. Circ: Cardiovasc Interv. (2024) 17(10):e014529. 10.1161/CIRCINTERVENTIONS.124.014529 39215512

[42] Colletti G Sgueglia GA Gasparini GL Ungureanu C Tsigkas G Leibundgut G Distal radial access for radial artery recanalization: multicenter outcomes and stepwise strategies to maximize patency. JACC: Cardiovasc Interv. (2025) 18(17):2140–51. 10.1016/j.jcin.2025.07.003 40930602

[43] Lin Y Bei W Liu H Liu Q Yuan J Wu M Retrograde recanalization of radial artery occlusion via the distal transradial artery: a single-center experience. Front Cardiovasc Med. (2022) 9:985092. 10.3389/fcvm.2022.985092 36211561 PMC9543139

[44] Wang H Cui C Liu H Zhang B Tian T Ye S Preliminary study on retrograde recanalization of radial artery occlusion through distal radial artery access: a single-center experience. Cardiovasc Drugs Ther. (2024) 38(6):1303–13. 10.1007/s10557-023-07490-9 37498472 PMC11680607

[45] Achim A Kakonyi K Jambrik Z Olajos D Nemes A Bertrand OF Distal radial artery access for recanalization of radial artery occlusion and repeat intervention: a single center experience. J Clin Med. (2022) 11(23). 10.3390/jcm11236916 36498491 PMC9740525

