# Supplementary material for: Radial artery thrombus remodeling after transradial access: from fresh components to recanalized channels on OCT
Source: Front Cardiovasc Med. 2026 Apr 14;13:1811724. doi: 10.3389/fcvm.2026.1811724 (PMC13120938; doi:10.3389/fcvm.2026.1811724)
Supplement: Supplementary file 1 [file Datasheet1.pdf]

**Supplementary Figure S1. Guidewire artifact and its effect on thrombus imaging**

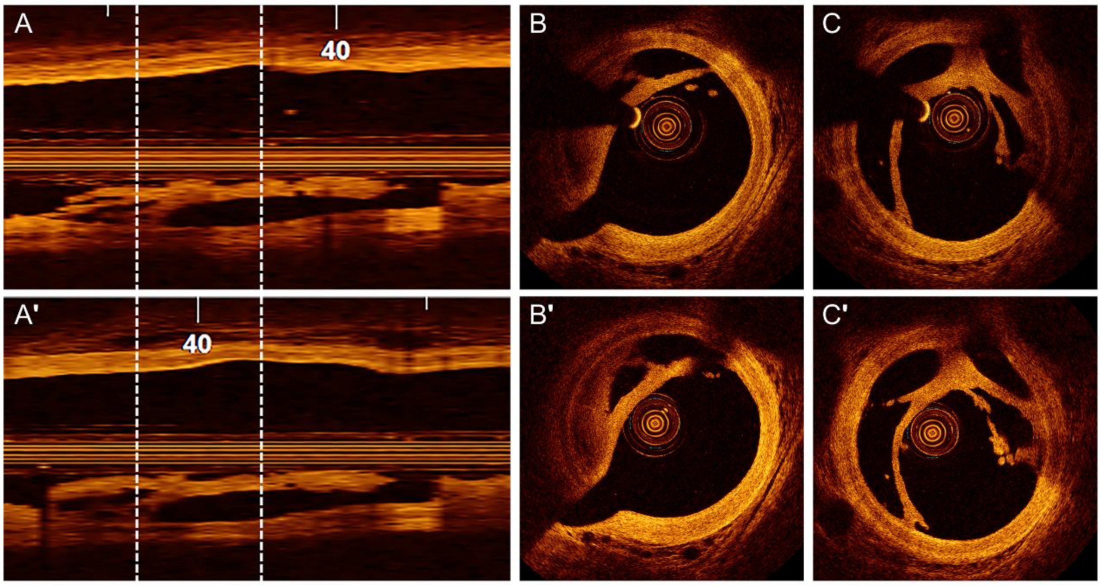


(A) Guidewire artifact in the longitudinal cross-section. (A′) Corresponding segment without guidewire artifact. (B) Guidewire artifact arc of 30.3°, partially obscuring the thrombus channel, as shown in (B′). (C) Guidewire artifact arc of 28.7°, with septa obscured, clarified in (C′).

**Supplementary Figure S2. Registration and determination of analyzable pullback length in off-wire OCT imaging.**

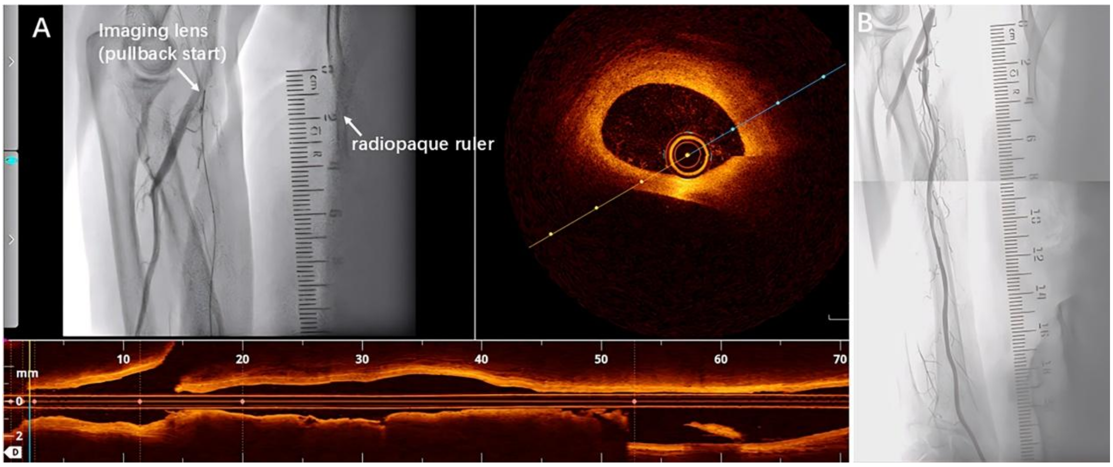


(A) Fluoroscopic image showing the OCT imaging lens at the pullback start point, aligned with a radiopaque ruler placed along the radial artery. The corresponding cross-sectional OCT image represents the vessel lumen at the position of the imaging lens, and the longitudinal OCT view identifies the same point as the start of the analyzed pullback segment.

(B) Radial artery angiography with the radiopaque ruler serving as an external reference. The ruler enabled spatial correspondence between angiographic anatomy and the OCT pullback start point identified in panel A, allowing accurate determination of the analyzable segment

and preventing duplicate analysis of overlapping regions.

**Abbreviation:** OCT, optical coherence tomography.

**Supplementary Figure S3. Schematic illustration of procedurally defined radial artery segments for OCT analysis.**

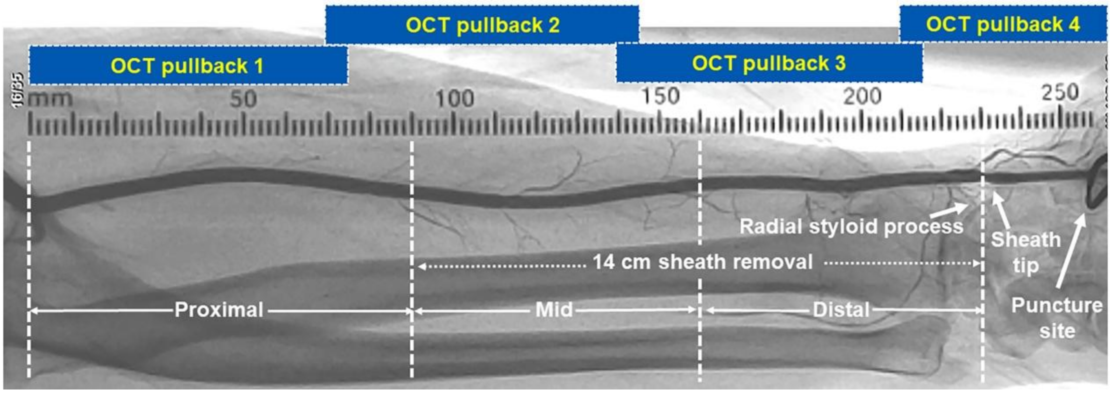


External and angiographic landmarks were used to define the spatial anatomy of the analyzed radial artery. A radiopaque ruler referenced the artery from the radial ostium, and a towel clamp placed at the skin entry site identified the puncture location. After sheath retraction, the in-artery sheath length was approximately 14 cm and was divided into 2 equal sheath-present segments (mid and distal, 7 cm each). Accordingly, the radial artery was divided into 3 procedurally relevant segments: proximal (non-sheathed), mid (sheath-present), and distal (sheath-present and puncture-related).

**Supplementary Figure S4 Radial Artery Occlusion with Recanalization Assessed by Angiography and Optical Coherence Tomography**

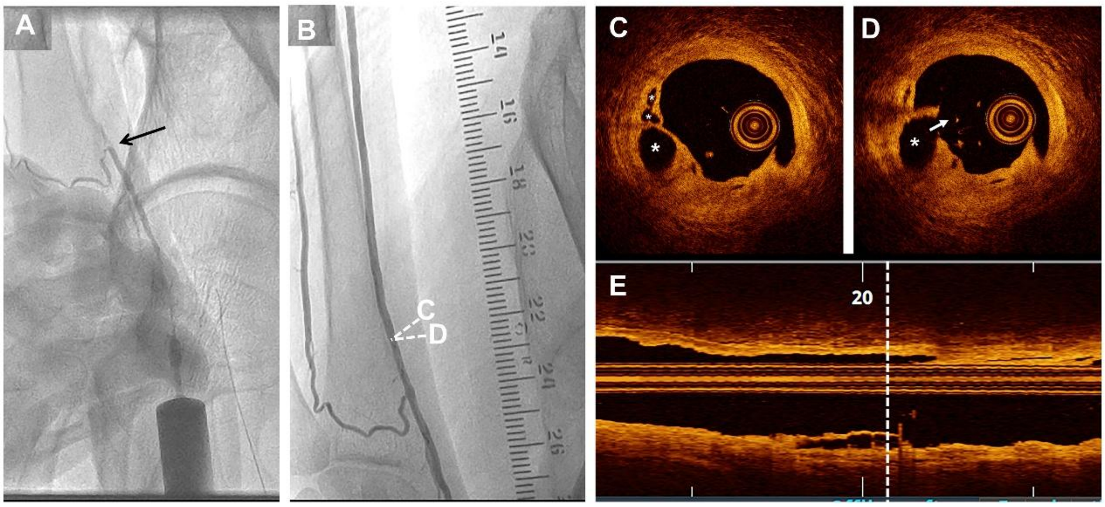


(A) Radial angiography demonstrating total occlusion (arrow). (B) Angiography after OCT pullback showing recanalization; dashed lines indicate the levels corresponding to panels C and D. (C) OCT cross-section demonstrating a recanalized thrombus with 3 intrathrombus channels (asterisks). (D) One channel communicates with the parent lumen (arrow). (E) Longitudinal OCT view corresponding to panels C and D.

**Abbreviation:** RAO, radial artery occlusion; OCT, optical coherence tomography.
