# Supplementary material for: Radial artery thrombus remodeling after transradial access: from fresh components to recanalized channels on OCT
Source: Front Cardiovasc Med. 2026 Apr 14;13:1811724. doi: 10.3389/fcvm.2026.1811724 (PMC13120938; doi:10.3389/fcvm.2026.1811724)
Supplement: Supplementary file 2 [file Datasheet2.pdf]

Supplementary Table S1. Technical Details of Retrograde Recanalization and Post-Recanalization OCT Findings in 4 Cases of Angiographically Confirmed Radial Artery Occlusion

Case	Time Since Most Recent Ipsilateral TRA, Days	Initial Crossing Wire	Polymer-Jacketed Wire Used Initially	Wire Escalation Required	Escalation Wire	Presumed Microchannel Crossing	Knuckle Technique	Adjunctive Lumen Modification	Post-Recanalization OCT Phenotype
1	39	Pilot 50	Yes	No	None	Yes	Yes	2.0-mm balloon predilatation	Type 1 + 2b
2	43	Pilot 50	Yes	No	None	Yes	Yes	Balloon-assisted crossing	Type 1 + 2b
3	45	Fielder XTR	Yes	Yes	Pilot 50	Yes	Yes	Balloon-assisted crossing	Type 1 + 2a
4	1699	GAIA Second	No	Yes	Pilot 50	Yes	Yes	Balloon-assisted crossing	Type 2a + 3

Supplementary Table S2. Interval-Stratified Thrombus Phenotypes by Time Since Last Transradial Procedure (Type-Based)

Variable	Overall (N=18)	≤30 days (n=1)	31–180 days (n=8)	181–365 days (n=2)	>365 days (n=7)	P (Exploratory)	Test	N Used
Days since last TRA, days	182 [44–1418]	2 [2–2]	44 [38–48]	266 [235–298]	1786 [1270–3308]	—	— (stratification variable)	18
Any thrombus (any type), n (%)	18 (100.0)	1 (100.0)	8 (100.0)	2 (100.0)	7 (100.0)	—	— (all 100%)	18
Any Type 1 thrombus, n (%)	14 (77.8)	1 (100.0)	8 (100.0)	2 (100.0)	3 (42.9)	0.011	Cochran–Armitage trend	18
Any Type 2a thrombus, n (%)	3 (16.7)	0 (0.0)	2 (25.0)	0 (0.0)	1 (14.3)	0.762	Cochran–Armitage trend	18
Any Type 2b thrombus, n (%)	7 (38.9)	0 (0.0)	5 (62.5)	0 (0.0)	2 (28.6)	0.395	Cochran–Armitage trend	18
Any Type 3 thrombus, n (%)	7 (38.9)	0 (0.0)	0 (0.0)	2 (100.0)	5 (71.4)	0.004	Cochran–	18

							Armitage trend	
Maximum channels (Type 3 only)	6.5 [4.2–8.0]	—	—	5.5 [4.2–6.8]	5.0 [5.0–8.0]	0.693	Mann–Whitney U (181–365 vs >365)	7 (Type 3+)
Total thrombus length (sum across segments/types), mm	48.3 [29.4–105.0]	29.0 [29.0–29.0]	117.4 [69.5–191.4]	48.5 [42.5–54.5]	18.8 [13.6–48.3]	0.053	Kruskal–Wallis	18
RAO at enrollment, n (%)	4 (22.2)	0 (0.0)	3 (37.5)	0 (0.0)	1 (14.3)	0.469	Cochran–Armitage trend	18

Values are median [interquartile range] or n (%). Exploratory P values are unadjusted for multiple comparisons. Type definitions: Type 1 = fresh/early; Type 2a = nonrecanalized without cavities; Type 2b = nonrecanalized with intrathrombus cavities; Type 3 = recanalized with communicating channels. Cochran–Armitage tests evaluate monotonic trends across ordered time intervals. Channel-related metrics are reported only for participants with Type 3 thrombus. RAO = radial artery occlusion; TRA = transradial access.

Supplementary Table S3. OCT-Based Morphologic and Quantitative Characteristics by Segment

OCT Variables	Overall (N=18)
**Proximal segment**	
Reference diameter, mm	2.81 ± 0.44
Reference area, mm²	6.36 ± 1.92
Type 1 (fresh/early), present, n (%)	6 (33.3)
Length, mm	20.00 [11.25–32.88]
Minimum lumen area (MLA), mm²	2.55 [2.23–3.93]
Area stenosis, %	57.20 [43.76–65.73]
Type 2a (nonrecanalized; no cavities), present, n (%)	2 (11.1)
Length, mm	40.50 [38.25–42.75]
Minimum lumen area (MLA), mm²	4.58 [4.48–4.69]
Area stenosis, %	42.96 [41.11–44.81]
Type 2b (nonrecanalized; cavities), present, n (%)	2 (11.1)
Length, mm	50.00 [40.00–60.00]
Minimum lumen area (MLA), mm²	2.79 [2.77–2.82]
Area stenosis, %	60.41 [59.88–60.94]
Type 3 (recanalized), present, n (%)	2 (11.1)
Length, mm	2.50 [2.25–2.75]
Minimum lumen area (MLA), mm²	3.96 [3.83–4.08]
Area stenosis, %	26.60 [25.08–28.13]
Maximum channels, n	5.50 [4.25–6.75]
Mean channels per segment	1.80 [1.56–2.05]
Sum of channel areas, mm²	0.20 [0.17–0.22]
Maximum channel area, mm²	0.60 [0.54–0.66]

Min/Max channel area ratio	4.12 [3.69–4.56]
Mean septa thickness, µm	160 [150–170]
**Mid segment**	
Reference diameter, mm	2.66 ± 0.23
Reference area, mm²	5.67 ± 0.88
Type 1 (fresh/early), present, n (%)	8 (44.4)
Length, mm	16.00 [11.12–37.75]
Minimum lumen area (MLA), mm²	3.56 [3.22–4.06]
Area stenosis, %	33.65 [26.82–41.00]
Type 2a (nonrecanalized; no cavities), present, n (%)	2 (11.1)
Length, mm	51.00 [46.50–55.50]
Minimum lumen area (MLA), mm²	3.71 [3.68–3.73]
Area stenosis, %	38.82 [36.66–40.98]
Type 2b (nonrecanalized; cavities), present, n (%)	4 (22.2)
Length, mm	35.10 [30.10–44.20]
Minimum lumen area (MLA), mm²	4.38 [3.81–4.74]
Area stenosis, %	30.00 [23.14–38.83]
Type 3 (recanalized), present, n (%)	4 (22.2)
Length, mm	14.00 [11.25–16.00]
Minimum lumen area (MLA), mm²	3.32 [3.17–4.04]
Area stenosis, %	21.29 [19.23–25.33]
Maximum channels, n	4.50 [3.75–5.75]
Mean channels per segment	1.54 [1.44–1.71]
Sum of channel areas, mm²	0.46 [0.42–0.50]
Maximum channel area, mm²	1.35 [1.25–1.43]
Min/Max channel area ratio	7.29 [4.50–9.81]

Mean septa thickness, µm	180[140–220]
**Distal segment**	
Reference diameter, mm	2.64 ± 0.29
Reference area, mm²	5.54 ± 1.28
Type 1 (fresh/early), present, n (%)	12 (66.7)
Length, mm	15.95 [11.55–29.50]
Minimum lumen area (MLA), mm²	4.02 [3.34–4.24]
Area stenosis, %	27.82 [25.60–49.04]
Type 2a (nonrecanalized; no cavities), present, n (%)	3 (16.7)
Length, mm	33.60 [20.80–52.80]
Minimum lumen area (MLA), mm²	3.74 [3.66–4.12]
Area stenosis, %	18.70 [11.38–29.20]
Type 2b (nonrecanalized; cavities), present, n (%)	7 (38.9)
Length, mm	30.20 [21.20–39.50]
Minimum lumen area (MLA), mm²	4.28 [3.99–4.56]
Area stenosis, %	25.18 [15.15–46.22]
Type 3 (recanalized), present, n (%)	5 (27.8)
Length, mm	8.6 [2.0–11.75]
Minimum lumen area (MLA), mm²	4.09 [4.04–4.55]
Area stenosis, %	15.21 [9.62, 19.70]
Maximum channels, n	3.00 [3.00, 5.00]
Mean channels per segment	1.33 [1.20, 4.07]
Sum of channel areas, mm²	0.32 [0.31, 0.47]
Maximum channel area, mm²	0.86 [0.74, 1.19]
Min/Max channel area ratio	14.00 [8.92, 23.50]
Mean septa thickness, µm	120 [110, 140]

Values are mean ± SD, median [interquartile range], or n (%). Channel-related metrics are

reported only for segments with recanalized (Type 3) thrombus. MLA = minimal lumen area; OCT = optical coherence tomography; TRA = transradial access.

Supplementary Table S4 Morphologic Characteristics of Recanalized (Type 3) Thrombus by Segment

Variable	Proximal	n	Mid	n	Distal	n	P Value
Mean channels	1.8 [1.6–2.1]	2	1.5 [1.4–1.7]	4	1.3 [1.2–2.0]	5	—
Total channel area, mm^2	0.20 [0.17–0.22]	2	0.46 [0.42–0.50]	4	0.32 [0.31–0.47]	5	—
Mean septa thickness, µm	160 [155–165]	2	185 [140–222]	4	120 [110–140]	5	—

Values are median [interquartile range]. Given the limited number of observations, comparisons are descriptive and no formal statistical testing was performed. OCT = optical coherence tomography.
